# A critical analysis of the current South African occupational health law and hearing loss

**DOI:** 10.4102/sajcd.v67i2.694

**Published:** 2020-03-24

**Authors:** Warren G. Manning, Mershen Pillay

**Affiliations:** 1 Discipline of Audiology, School of Health Sciences, University of Kwazulu-Natal, Durban, South Africa; 2 Discipline of Speech-Language Pathology, School of Health Sciences, University of Kwazulu-Natal, Durban, South Africa

**Keywords:** chemical, ototoxicity, occupational health, occupational health and safety law, audiology, hearing loss

## Abstract

**Background:**

Occupational health laws must recognise the constitutional requirement of substantive equality, and its role in ‘the progressive realisation’ of the rights provided by Section 27.

**Objectives:**

Our main aim is to review current South African occupational health law (vis-à-vis workers’ constitutional rights) in relation to hearing loss. We focus on gaps in the law regarding occupational hearing loss in South Africa.

**Method:**

Our review of legal texts relies on experience as a methodological device augmented by the use of a critical science. Guided by literature or evidence synthesis methodologies, South African primary and secondary laws were reviewed along with unpublished (non-peer-reviewed) grey literature. An established six-step framework guided our thematic analysis. A semantic approach aided the critical interpretation of data using the Bill of Rights as a core analytical framework.

**Results:**

Four themes are discussed: (1) separate and unequal regulatory frameworks; (2) monologic foregrounding of noise; (3) minimisation of vestibular disorders; and (4) dilution of ototoxic agents. The highly divided legal framework of occupational health and safety in South Africa perpetuates a monologic ‘excessive noise-hearing loss’ paradigm that has implications for the rights of all workers to equal protections and benefits. There is a need to harmonise occupational health and safety law, and expand the scope of hearing-protection legislation to include the full range of established ototoxic hazards.

**Conclusion:**

Occupational audiology is dominated by efforts to address noise-induced hearing loss. A ‘noise’ despite the reality of workers’ exposures to a range of ototoxic stressors that act synergistically on the ear, resulting in audio-vestibular disorders.

## Introduction

In this review, we present an understanding of occupational hearing loss (OHL) in relation to South African health law. Traditionally, a review of any health condition would consist of an epidemiological description augmented with statistics like the size of the affected populations, incidence and prevalence rates, morbidity and mortality rates, and estimated economic burdens and other costs. However, the literature on OHL is characterised by a lack of data. This notch in epidemiological healthcare literature is an artefact of audiology’s history as a healthcare practice, particularly in South Africa (Osewe & Nkrumah, 2018).

**FIGURE 1:**
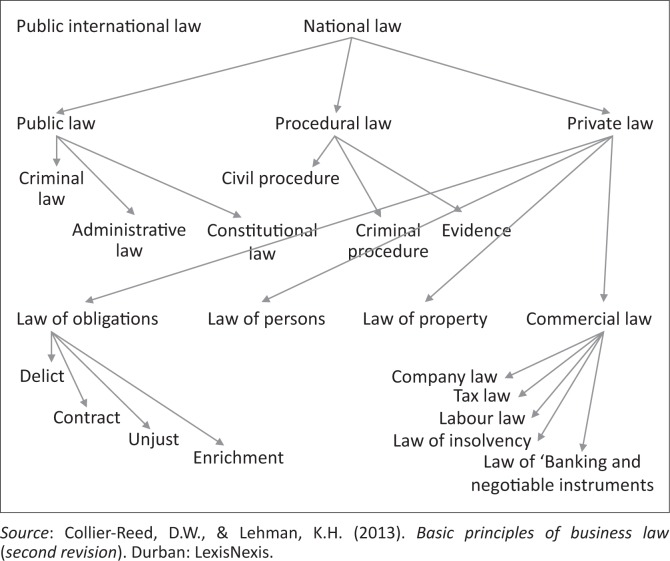
Branches of South African Law.

**FIGURE 2:**
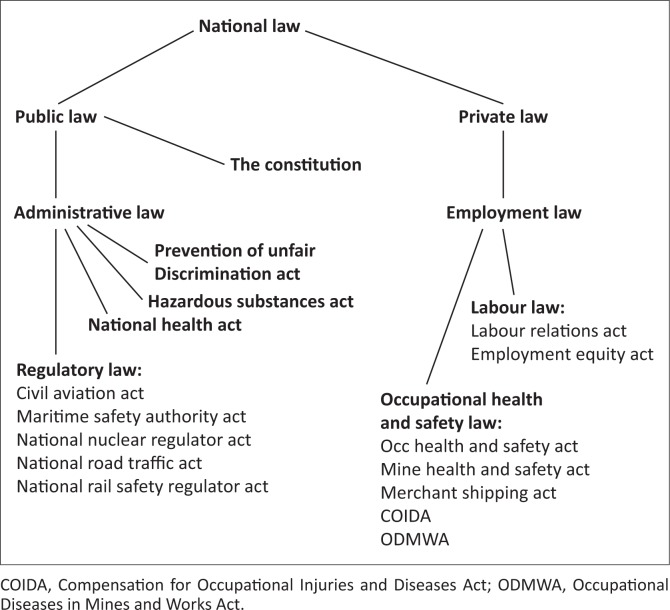
Structure of South African Law.

Our raison d’être is entwined with the Constitution in Chapter Two of the South African Bill of Rights, which outlined standards for equality and the provision of healthcare services that all subordinate law, policy and administrative actions must meet. Section 9 mandates the state to provide equal protection and benefit to all and disallows unfair direct and indirect discrimination. Further, section 27 mandates the state to progressively realise healthcare rights (South African Government, 1996). In order to understand the functional nature of law, it is first necessary to appreciate the general structure of South African legislation as was illustrated by Collier-Reed and Lehman (2013) (Figure 1):

Occupational healthcare is implicit in public law via regulatory laws and explicit in private law in the employment law sub-branch via the occupational health and safety laws. There are at least 11 laws, within public law (administrative law) and private law (commercial law) branches, requiring occupational healthcare services. In addition, there are regulations, guidelines and codes of practice that expand the number of legally binding requirements. The structure of South African law can therefore be overlaid by laws relevant to our focus, as demonstrated in Figure 2.

There exists a complex legal framework (see Appendix 1) that spans general occupational sectors and specific mining, maritime, aviation, railway, transport and nuclear or energy sectors. The regulations and codes of practice associated with these sectors may refer to workers’ balance and/or hearing functions. Notably, chemicals in the workplaces may be ototoxic (Nakhooda, 2016) or, when combined with noise, exacerbate hearing loss (Moroe & Khoza-Shangase, 2018). Therefore, acts and codes for hazardous substances also bear relevance to OHL. Furthermore, South Africa has two worker compensation acts, namely, *Compensation for Occupational Injuries and Diseases Act* (COIDA) and the *Occupational Diseases in Mines and Works Act* (ODMWA). These acts differ in the administration and benefits provided – and indication of these legal and worker compensation frameworks encode or reflect theoretical understandings of what constitutes a hearing loss in the workplace, what etiological factors are responsible for OHL as well as how to measure and manage workers’ hearing. Indeed, and has been previously argued (Pillay, 2001, 2011a; Pillay & Kathard, 2018), such understandings are usually unclear, hidden and not well-understood by audiologists outside of a few basic references to South African National Standard (SANS) guidelines or COIDA. Significantly, this has an impact on worker rights when we consider that these are laws that mandate occupational hearing protections. The question this raises is: how adequately, in this legal quagmire of acts and regulations, is the state realising South African worker rights as per Sections 9 and 27 of the Bill of Rights? Therefore, the main aim of this article is to review current South African occupational health law (vis-à-vis workers’ constitutional rights) in relation to hearing loss. This review focusses on the identification of gaps in the law regarding OHL in South Africa. The intention is to highlight the nature of work, the challenges and opportunities that hearing healthcare professionals – and specifically audiologists – need to pay attention to in post-1994 South Africa.

## Methodology

This review is based on our combined experience of over 50 years in occupational health and audiology. Experience used as a methodological device (Daher, Carré, Jaramillo, Olivares, & Tomicic, 2017) is not a thoughtless method lacking theory. Instead, we rooted experience within an old (but relevant) framework proposed by Pillay, Kathard and Samuel (1997) who argued for South African audiologists’ use of a critical science to re-look at inequities. As such, we reflexively reviewed laws with social, political, cultural, gendered, historical and allied contexts related to how knowledge of workers’ hearing has been produced. Methodologically, this facilitated constructing, deconstructing (cf decolonising: Pillay & Kathard, 2015, 2018) and re-constructing new meaning across occupational law. We relied on the inherently subjective (González Rey & Mitjáns Martínez, 2019:21) and useful method of dialogue or critical conversations (Pillay, 2011b). Finally, we read these laws within a political framework (Kathard & Pillay, 2013) to interpret worker rights.

While not designed as a literature review study, *per se*, we were influenced by evidence synthesis methodologies to review South African primary and secondary law. The first author, an occupational health and safety expert, identified relevant acts, regulations and codes of practice. Selected for their current relevance in South African law, these laws refer to various occupational sectors, and they contain references to noise, hearing, vestibular disorder, dizziness, vertigo, giddiness, nystagmus, Ménière’s disease, radiation, pressure and barotrauma. Notably, we excluded laws applicable to the South African National Defence Force and the South African Police Services because of their unique legislation and compensation laws. We identified a lot of grey literature (unpublished or non-peer-reviewed legal documents) to enrich our analysis. We made sense of what we read using Braun and Clarke’s (2006) six-step framework. We familiarised ourselves with the data, followed by a system of coding of selected text towards identifying themes that were then clustered for the development of a conceptual map. We further identified thematic patterns and identified textual extracts. A semantic approach was followed in order to describe the content of current legal data. We then critically interpreted possible implications to the rights of all persons for equal protection and benefit from the law – cross referencing the Bill of Rights as a core analytical framework.

### Ethical considerations

The authors confirm that ethical clearance was not needed or required for this study.

## Results and discussion

We identified the following major themes:

- separate and unequal regulatory frameworks
- monologic foregrounding of noise
- minimisation of vestibular disorders
- dilution of ototoxic agents.

### Separate and unequal regulatory frameworks

Occupational Health and Safety (OHS) law is fragmented based on scopes and types that mandate employee’s medical monitoring. Firstly, the division in scope spans three occupational fields: general, mining and shipping – with shipping being further divided, wherein cargo handlers (e.g. stevedores) are covered under the *Occupational Health and Safety Act* (OSHA) (Republic of South Africa, 1993b) and all other personnel on the ship are covered under the *Maritime Shipping Act* (MSA) (Republic of South Africa 1951). Therefore, different legislative requirements apply to each group. It must be noted that the MSA dates from the 1950s and lacks an analogous structure and purpose as compared to the OHSA and the *Mine Health and Safety Act* (MHSA). The MSA mainly has a regulatory purpose to manage, for example, licensing and labour relations.

A second division between the types of law resulted in the separation of regulatory and employment law. The three OHS laws fall within the employment law division, but the laws creating the specific regulator bodies, for example, the Civil Aviation Authority (CAA) falls under regulatory law. While occupational health and safety law is focussed on the creation of safe and healthy work environments, regulatory law is focussed primarily on liability management, and it can be argued that these laws address occupational illness from a tort law perspective where illness is characterised as a result of ‘human error’ (Hutchings, 2017), a risk that must be managed.

In this regulatory and employment division, the following characteristics are critical: (1) codes of practice under OHSA and MSA have a different status compared to the codes of practice under MHSA and (2) the use of exposure limits from other jurisdictions, for example, from the United Kingdom occur – as in the case of the Maritime sector (2007). There are several other characteristics that make the set of relevant legal frameworks unequal, namely:

- A variety of nomenclature to designate and empower medical practitioners, not all of whom are defined as occupational medical practitioners, for example, designated aviation medical practitioner (Republic of South Africa, 2001; South African Civil Aviation Authority, 2011) and occupational health practitioner (Republic of South Africa, 2000a).
- Reporting mechanisms differ; for example, in MSA, as per s5(e), the ‘employer’ alone reports cases (1996), or in shipping, a case is entered into the ships’ official log-book and, as per s185 of MSA, the Master delivers this log-book to the ‘proper officer’ when the ship returns to port (1996).
- Regulatory bodies focus on liability management while the impact of working conditions appears to be minimised.
- Different and uncomplimentary organisational cultures persist; for example, in aviation (SA-CATS, 2011: CAA, Schedule 28: Obstetrics and Gynaecology), this includes ‘menstrual disturbances’ and ‘medical requirements following confinement or termination of pregnancy’ (2011). The use of an irregular term (menstrual disturbance) and the inclusion of outdated traditional practices (confinement) are both remnants of a past paternalistic culture.
- Medical standards exist with highly detailed protocols (e.g. aviation) or as accepted standards (maritime, railways) that manage ‘human factors’ as risk sources or, as dictated by organisational culture, used interchangeably with human error (Hutchings, 2017), giving no cognisance to ill health from occupational stressors. Critically, these medical standards do not provide instructions on rehabilitation protocols.
- Hazardous Biological Agents Regulations (2001), Hazardous Chemical Substances Regulations (HCSR) (1995) and the Lead Regulations (2001) contain medical surveillance standards but exclude reference to ototoxicity in spite of known ototoxic agents, for example, infectious agents, solvents and lead. National Road Traffic Regulations (Republic of South Africa, 2000a) and Construction (2014) require medical certification without any medical fitness standards. Both work environments contain known ototoxic agents like carbon monoxide and solvents.
- Medical surveillance and medical incapacity management protocols are unequal; for example, MHSA is unmatched for its promulgation of standards for exposure, fitness, medical surveillance implementation and management of rehabilitation and return to work.
- Sector disparities exist with workers in noisy workplaces benefitting from legislation (e.g. the Noise-Induced Hearing Loss Regulations [NIHLR]) to manage this specific ototoxic agent without similar benefits for those exposed to ototoxic agents, such as chemicals, excessive pressure changes or radiation.

In summary, South African OHS law is highly fragmented and has been so for over the last 40 years, as commented on by Ncube and Kanda (2018). They explained that these fragmentations have been resulting in duplication of law enforcement roles; a costly waste of scarce resources; and inconsistencies and lack of uniformity in the implementation of enforcement functions. The main outcome is a hampering of progress at safeguarding workers’ health. Our analysis is aligned with Ncube and Kanda’s conclusions that there are four departments (labour, mining, transport and health) creating legislation and five regulatory bodies (aviation, rail, road, nuclear, maritime) creating specific management protocols. The shipping sector does not have any pure occupational health and safety enabling legislation equivalent to the OHSA and MHSA. Overall, it is therefore not surprising that systemic inconsistencies exist for the provision of occupational audiology protections and services in South Africa.

The next three sections, summarised in Table 1, focus on more specific issues:

**TABLE 1:** Summary of the core themes emerging from an analysis of South African General, Mining and Shipping Acts and regulations of occupational hearing loss.

Theme	Sector		
	General	Mining	Shipping
Monologic Foregrounding of Noise	NIHL: Exposure to noise noise limit ‘noise zones’ CI 171	Schedule 22.9(2)(b)(i) Noise limit Guidelines: Occupational Health Programme 2003 (Occupational Hygiene and Medical Surveillance) for noise Implementation of Standard Threshold Shift in The Medical Surveillance of Noise- Induced Hearing Loss 2016 CI 171	Codes and NIHLR: Nose limit ‘check the noise level’ ‘ear protection’ ‘soundproofing of workstations’ CI 171
Minimisation of vestibular disorders	Diving: inner ear involvement, other systems of the body, and splinting the tympanic membrane, etc. Specific diving injuries and diseases Chemicals may cause systemic effects Rail: Meniere’s Disease Aviation: Vestibular neuronitis (and acute labyrinthitis), Menière’s disease, benign paroxysmal position nystagmus and other miscellaneous causes of vertigo should be taken into account and applicants assessed accordingly	11.4 Noise medical surveillance contemplated in regulation 11.4(1) must consist of a baseline audiogram, periodic audiograms and an exit audiogram	Merchant Shipping (Eyesight And Medical Examination) Regulations, 2004: Disabling Meniere’s Disease - Category B
Dilution of other ototoxic stressors	Diving: Radiation hazards, noise, temperature extremes and pressure Pressure (causing barotrauma, decompression sickness) pulmonary barotrauma	Regulation 9: hazards that may cause illness or adverse health effects to persons no employee is exposed to any health hazard	Codes: ‘gasses, fumes, dust, radiation, excessive noise’ ‘harmful to face and eyes’ ‘potential cause of skin damage’

NIHLR, Noise-Induced Hearing Loss Regulations.

### Monologic foregrounding of noise

The current legal doctrine mandating occupational audiology protection is essentially monologic, as its theoretical rationality is centred on ‘excessive noise’ as the *only* ototoxic stressor and ‘hearing loss’ as the only negative health outcome. For example, NIHL regulation 3 includes exposure to noise and sets the noise exposure limit at 85 dBA (Republic of South Africa, 2003); regulation 8 sets the requirements for a system of environmental monitoring and medical surveillance of ‘noise zones’ (Republic of South Africa, 2003). Similarly, the title of SANS 10083 (South African Bureau of Standards, 2013) is clear about its focus, namely, ‘The measurement and assessment of occupational noise for hearing conservation purposes’ as is the Compensation Commissioner’s Circular Instruction No. 171, entitled ‘Determination of Permanent Disablement Resulting from Noise-Induced Hearing Loss and Trauma’. Similarly, the Diving Regulation Codes of Practice (South African Department of Labour, 2001) cite ‘noise’ approximately eight times and ‘hearing’ or ‘hearing loss’ four times in its text. Chemical and pressure hazards are noted but inconsistently referenced to audio-vestibular disorder risks. In mining, noise-induced hearing loss is prioritised by standards for pollutants, heat and noise (regulation 9, 1996) – specific guidelines for noise exposure management (Department of Mineral Resources, 2003b) and hearing conservation (Standard Threshold Shift) (Department of Mineral Resources, 2016a). The fitness standard uses a pure tone audiometric assessment protocol (Fitness Standards) (Department of Mineral Resources, 2003a). This monologic rationality is echoed in shipping with the MSA and *South African Maritime Safety Authority Act* (SAMSA) (Republic of South Africa, 1998a) whose regulations and codes of practice emphasise the role of noise and the importance of noise control by focussing on an insistence by Portnet (1994) that one ‘must check the noise level’ alongside the use of ear protection and noise exposure (Maritime and Coastguard Agency [MCA], 2018) or the reduction of noise levels and soundproofing of workstations (MCA, 2018).

The COIDA, by its presumption in Section 66, recognises that disease conditions listed in Schedule 3 are because of an occupational overexposure for which this insurance regime considers compensation. However, ‘hearing loss caused by excessive noise’ is the only specific audio-vestibular disability or disease that is listed.

In summary, the dominant narrative is one of the need to control ‘noise’ exposure to prevent ‘hearing-loss’. It is well-established that chemicals, for example, complicatedly affect workers hearing in the presence of noise (Watts, 2019). Other stressors (pressure, heat, vibration, etc.) also complicate hearing loss (Lie et al., 2016). However, even if noise is the single stressor, then auditory effects are not the only outcome. Suicide, depression, anti-social behaviours – other psychological, social and even associated physiological (non-auditory) effects have disabling, if not fatal, outcomes on workers’ (and their families or social) lives (Basner et al., 2014). Thus, the monologic rationality that dominates the legal discourse results in a violation of worker rights by being selectively protective. This means that these laws indirectly discriminate worker rights. Indirect discrimination is the violation of one’s rights to equal protection because the existing policy disfavours a particular group, without justification. The analysis of the current legal doctrine normalises noise-induced hearing-loss as the standard occupational disability that must be prevented by the measures prescribed by the NIHLR as an example.

### The minimisation of vestibular disorders

None of the three legal frameworks address vestibular disorder in any substantial manner, save its inclusion in specific medical testing regimes. Aviation (South African Civil Aviation Authority, 2011), maritime (South African Maritime Safety Authority, 2016) and railway management specification (Southern African Railway Association [SARA], 2011) include requirements and protocols for the assessment of vestibular disorder – with a frequent reference to ‘Meniere’s Disease’, which minimises the recognition of occupational ototoxic agents. In mining, for example, in MHSA regulations 11 3 c (viii), broad statements exist like: ‘…(o)occupational diseases, past or present, including severity’ (Republic of South Africa, 1996) without specific reference to vestibular disorders. Mining regulations prescribe exclusive use of pure-tone audiometry, which is not suitable for detecting vestibular disorder. Similarly, diving documentation, such as the Code of Practice for Off-shore Diving (Department of Labour, 2017), includes statements like ‘…other systems of the body’ and ‘(s)pecific diving injuries and diseases’, which may beinterpreted as including audio-vestibular disorders. In reference to water pressure in the external ear canal, outcomes are noted as ‘…splinting the tympanic membrane, etc.,…’,where vestibular disorders may be included in the ‘etc.’. For COIDA, Schedule 3 (Republic of South Africa, 1993c) lists the wider range of audio-vestibular disorder within broader statements such as ‘…any disease or pathological manifestations…’ caused by the given list of chemical agents, ‘…dysbarism, including decompression sickness, baro-trauma or osteonecrosis,…’ and ‘…(a)ny disease caused by ionising radiation’. The term ‘ototoxic agent’ is not included.

In summary, vestibular disorders are rarely and barely recognised as occupational stressors, which results in selective medical testing, compromising worker rights – as discussed in the implications section.

### Dilution of ototoxic agents

Dilution of ototoxic agents refers to the grouping of agents together into a general hazard group. Only the SARA (2011) noted the ‘…(d)elayed effects such as nervous system toxicity, cancer of the lungs or chemically-induced hearing loss…’. The dominant doctrine places known ototoxic agents into a common basket of hazards, for example:

- The Hazardous Biological Agents Regulations (Republic of South Africa, 2001), HCSR (Republic of South Africa, 1995) and the Lead Regulations (Republic of South Africa, 2002a) contain medical surveillance standards but do not include any reference to ototoxic outcomes even though these regulations are aimed at known ototoxic agents, for example, infectious agents, solvents and lead. The HCSR require Safety Data Sheets to be prepared in a form described in Annexure 8 (Republic of South Africa, 1995); it is not a requirement for known ototoxic chemicals to be labelled as such.
- Similarly, in the mining sector, the MHSA (Republic of South Africa, 1996) notes that there are ‘…significant hazards or risks the employee was exposed to, such as dust, noise, radiation, chemical or other’.
- In the shipping sector ‘…gasses, fumes, dust, radiation, excessive noise…’ are clustered together (Portnet, 1994). Chemicals are noted as ‘harmful to face and eyes’ and can ‘cause skin damage’ (MCA, 2018).
- The Diving Regulations codes of practice (Department of Labour, 2017) that refer to ‘…Health hazards’ include ‘Physical hazards’ and these include ‘Radiation hazards, Noise, Temperature Extremes and Pressure (causing barotrauma, decompression sickness)’. Chemicals are highly referenced in the codes, and it is noted that ‘…(s)kin exposure may cause local effects (e.g. chemical burns) or may cause systemic effects due to absorption of the chemical’ (p. 13), but these references do not highlight their ototoxic potential. Barotrauma appears twice (2); once as ‘pulmonary barotrauma’ but aural or otic barotrauma is never indicated.

In summary, diluting ototoxic agents into a homogenous etiological ‘phenomenon’ disguises the specific nature of ototoxic agents. This clustering of aetiological factors has implications for what and how workers are compensated. The compensation procedure (stipulated by CI 171) and the absence of any specific reference to other forms of audio-vestibular disorder in Schedule 3 of the COIDA places persons with these disease conditions at a disadvantage. In addition, as none of the regulatory agencies’ medical examination protocols explicitly refer to the legal obligation of Sections 24 and 25 of the OHSA, cases may go unreported to the Compensation Commission. This is, in fact, the committing of an offense in terms of the OHSA. The insistence of a medically simplistic meme of ‘one stressor-one outcome’, rooted to its monologic rationality, is an artefact of biomedical, empirical science that simplifies the complexities of workers’ lived realities. The implications of this are discussed next.

### Implications of the ‘one-stressor, one-outcome’ paradigm and worker rights

Three aspects of the current paradigm require evaluation for (1) the provision of reasonable protection in the workplace; (2) how medical surveillance testing occurs; and (3) the compensation for occupational disease. From our analysis above, it becomes apparent that workers are (1) selectively protected; (2) offered selective medical testing; and (3) selectively compensated across occupations for their OHL or disability in South Africa. As noted above, when noise is – legally – the prime ototoxic agent, regulations violate worker rights by being selectively protective. Facilitated by a monologic rationality, the full range of occupational audio-vestibular disorder is Othered outside of noise-induced hearing loss. The lack of reform of the regulation, for example, NIHLR to include other known ototoxic agents is contrary to the mandate to progressively extend protection to all employees including those in non-noisy but oto-traumatic occupations.

Medical testing is then cast as selective. Consider (*Employment Equity Act* [Section 7] [Republic of South Africa, 1998b]):

(1) Medical testing of an employee is prohibited, unless … it is justifiable in the light of medical facts, employment conditions, social policy, the fair distribution of employee benefits or the inherent requirements of a job. (n.p.)

Medical surveillance and fitness testing that exclude protocols to determine non-noise audio-vestibular disorders or disabilities are prohibitive. They do not recognise established medical facts regarding the polylogical aetiologies of occupational audio-vestibular disorder.

Selective medical testing is a healthcare mechanism that prohibits the fair distribution of employee benefits and does not acknowledge the inherent requirements of a job. Such medical testing implies that workers are selectively compensated, indicating a form of worker discrimination. Under the *Promotion of Equality and Preventi on of Unfair Discrimination Act* (Republic of South Africa, 2000b), discrimination is defined as:

[*A*]ny act or omission, including a policy, law, rule, practice, condition or situation which directly or indirectly-

- imposes burdens, obligations or disadvantage on; or
- withholds benefits, opportunities or advantages from any person on one or more of the prohibited grounds… (n.p.)

In Section 14, the ‘determination of fairness or unfairness’test includes a consideration of the social position of the affected persons and the systemic nature of the discrimination, its nature and purpose. A legal framework, as we have demonstrated, which inadvertently disadvantages or withholds benefits, such as insurance products, from reaching workers for scientifically unjustifiable reasons because of the manner in which the system of occupational health and safety operates will face a serious challenge to be accepted as *‘*fair’.

### Summary and recommendations

In summary:

- South African workers are exposed to a range of oto-traumatic agents, for example, noise, pressure, radiation in addition to background exposure to a growing list of chemical pollutants (e.g. Matatiele et al., 2019; Niranjan, 2015).
- Exposure to these agents (singularly or in combination) may likely lead to a range of audio-vestibular diseases not limited to auditory effects or loss of hearing acuity, per se.
- The loss of audio-vestibular function may lead to dismissal on grounds of medical incapacity.
- Current compensation laws will not necessarily acknowledge claims of non-noise-induced hearing losses.
- The exclusion of established hearing assessment protocols from the mandatory medical surveillance examinations is irrational and discriminatory.

We recommend:

- a critical review of all laws relevant to hearing healthcare (specifically, occupational audiology) intended to broaden the definition of OHL
- rationalisation and harmonisation of the OHS law into one system
- specific inclusion of audio-vestibular disorders under COIDA
- identification of gaps in OHL research such as epidemiological data
- development of adjusted standards for:
  ■ exposure limits including realistic combinations of ototoxic agents
  ■ environmental monitoring, for example, codes of practice for noise measurement in under-water and deep mining contexts
  ■ guidelines for the transportation of mine workers in deep-level mining.

## Conclusion

In 2016, revelations of a systemic cover-up of occupational disease in the Silicosis Trial (Nkala and Others v Harmony Gold Mining Company Limited 2016) resurrected the spectre of gross human rights abuse in the workplace. The South African Human Rights Commission (2016) conducted a National Hearing on Unfair Discrimination in the Workplace. The report on these hearings made the following findings:

- Significant advances and gains made in labour practices since 1994, but unfair discrimination still pervades the workplace
- There is a lack of understanding by key role-players as to the meaning and complexity of unfair discrimination in its entirety
- There is a lack of awareness and/or sufficient attention paid to other forms of systemic discrimination taking place in the workplace
- Many instances and specific manifestations of unfair discrimination continue to occur inconspicuously and remain largely unreported
- One of the biggest driving factors of unfair discrimination is a lack of awareness and information.

While this report deals with disability in the workplace, it overlooks: (1) impairments of occupational cause; (2) the fairness of medical fitness assessments; and (3) the need for a review of the OHS law, with special attention to broadening the definitions of occupational disease and the need to establish valid medical surveillance standards. Like all forms of cultural blindness, ours is deep and effort is required to address the gaps and weakness in our OHS law in order to ensure equality to all. This work is urgent noting what Ackermann stated in the Constitutional Court ruling in the National Coalition for Gay and Lesbian Equality and Others v Minister of Home Affairs and Others (CCT10/99) (1999) case for same sex rights ‘like justice, equality delayed is equality denied’.

## Appendix 1

BOX A1 Selected legal frameworks (acts, regulations and codes) of South African occupational sectors for worker hearing.

**Table:**

***The Occupational Health and Safety Act (OHSA) 85 of 1993***	
This act falls under the jurisdiction of the erstwhile Minister of Labour, and it details the minimal legal requirements for the majority of the occupational contexts within the South African economy.	
Section 8	mandates the employer to provide ‘…a working environment that is safe and without risk to the health…’ and further establish ‘…what hazards to the health or safety of persons are attached to any work which is performed’.
Section 13	‘Duty to inform’ every employee of ‘the hazards to his health and safety’.
The minister may make regulations regarding *inter alia*:	
Section 43	‘(vi) any matter regarding the biological monitoring or medical surveillance of employees;
	(vii) … the exposure of employees … to, hazardous articles, substances or organisms … including specific limits, thresholds or indices of or for such exposure;’	
Regulations relevant to an assessment of equality in access to occupational audio-vestibular healthcare include:	
**Noise-Induced Hearing Loss Regulations (2003)**	These are the principle hearing conservation regulations under OHSA and serve as the standard for the protection of hearing in most workplaces in the Republic of South Africa (Republic of South Africa, 2003).
**Environmental Regulations for Workplaces 1987**	These regulations do not make specific requirements with respect to occupational audiology but incorporate the following four codes of practice (Republic of South Africa, 1987): SANS 10083:2013 The measurement and assessment of occupational noise for hearing conservation purposes SANS 1451-1:2008 Hearing protectors Part 1: Ear-muffs SANS 1451-2:2008 Hearing protectors Part 2: Ear-plugs SANS 1451-3:2014 Hearing protectors Part 3: Ear-muffs attached to an industrial safety helmet
**Diving Regulations 2010**	These ‘…(r)egulations apply to all diving operations and all persons engaged in diving operations in the Republic of South Africa or the territorial waters thereof…’ (Republic of South Africa, 2010). The specific nature of health stressors divers are exposed to and medical examinations required are provided in four codes of practice; these are the: Inshore Diving Code of Practice (Department of Labour, 2017) Offshore Diving Code of Practice (Department of Labour, 2014b) Scientific Diving Code of Practice (Department of Labour, 2014c) Benign Diving Code of Practice (Department of Labour, 2014a)
A third group of regulations of the OHSA requires medical surveillance or medical fitness testing but does not include audiological aspects within the scope of their assessments, namely:	
	the Asbestos Regulations, 2001 (Republic of South Africa, 2002b). Construction Regulations, 2014 (Republic of South Africa, 2014b). Hazardous Biological Agents Regulations, 2001 (Republic of South Africa, 2001). Hazardous Chemical Substance Regulations, 1995 (Republic of South Africa, 1995). Lead Regulations, 2001 (Republic of South Africa, 2002a).
***Mine Health and Safety Act* (MHSA) 29 of 1996**	
This act falls under the Minister of Mineral Resources and requires ‘employers and employees to identify hazards and eliminate, control and minimise the risks relating to health and safety at mines’ (Republic of South Africa, 1996).	
Sections 11, 12 and 13	Requires that ‘(e)very manager must: identify the hazards to health or safety to which employees may be exposed and periodically review the hazards identified and risks assessed, conduct occupational hygiene measurement of hazards and establish a system of medical surveillance’.
s(5B) (Republic of South Africa, 1997)	*employer must notify the Principal Inspector of Mines of any accident or occurrence at a mine that results in (b) illness.*
**MHSA Regulations**	
**Regulation 9.2 Occupational Hygiene**	Sets the exposure limit for noise as given in Schedule 22.9(2)(b)(i) as: (1) Noise Exposure: 82 dBL Aeq, 8h
**Regulation 11. Occupational Medicine**	Requires an ‘Exit Certificate’ to be prepared, which includes information regarding the: ‘…(f) Biological monitoring results of the employee, and provides comments on abnormal results…’. ‘…(g) Occupational diseases previously incurred and current including severity…’
**MHSA Guidelines**	
*The Guideline for the Compilation of a Mandatory Code of Practice for an Occupational Health Programme 2003* (Occupational Hygiene and Medical Surveillance) *for noise* (Department of Mineral Resources, 2003b). *The Guideline for Mandatory Code of Practice on Minimum Standards of Fitness to Perform Work on a Mine* (Department of Mineral Resources, 2003a). *The Guideline for a Mandatory Code of Practice for the Management of Medical Incapacity owing to Ill-Health and Injury* (Department of Mineral Resources, 2016b). *The Guidance note for the Implementation of Standard Threshold Shift in The Medical Surveillance of Noise-Induced Hearing Loss* (Department of Mineral Resource, 2016a).	
***The Merchant Shipping Act, No. 57 of 1951***	
This act comes under the jurisdiction of the Minister of Transport (Republic of South Africa 1951).	
MSA Regulations and Guidelines have been promulgated that address hearing conservation specifically.	
Via s356 **The Maritime Occupational Safety Regulations (Republic of South Africa, 1994)**	Requires that every employer shall ‘…(g) ensure that every employee is aware of the hazards connected with any work to be performed, or machinery to be used by him and that he is conversant with the safety measures to be taken or observed to obviate such hazards…’. These regulations incorporate three codes of practice: The Code of Safe Working Practices for Merchant Seamen (Maritime and Coastguard Agency, 2018b). The Code of Safe Working Practice for the Construction and Use of Fishing Vessels (Maritime and Coastguard Agency, 2018a). The South African Ports Cargo Handling Code of Practice (Portnet, 1994)
**The Merchant Shipping (Eyesight and Medical Examination) Regulations (2004)**	Require medical surveillance of the crew, and the standards are provided by the ‘Maritime Medical Standards Code’ of the ‘Maritime Qualifications Code’ (2004), which was adopted in the amendments of 2015.

MCA, Maritime and Coastguard Agency.

**TABLE A1:** Summary of hearing protection laws.

Sector	Act	Regulations	Exposure limit *LAeq* (8 h)	Code of practice		Notes
General	OHSA	Noise-Induced Hearing Loss Regulations 2003	85	*SANS 10083:2013*		For ‘;measurement and assessment of occupational noise …’
				*CI 171*		> 10% hearing loss; only ‘occupational disease caused by excessive noise’ must prove excessive noise.
Mining	MHSA	Mine Health And Safety Regulations 1996	*82*	Guideline For The Compilation of A Mandatory Code of Practice for An Occupational Health Programme 2003 (Occupational Hygiene and Medical Surveillance) FOR NOISE		*Follows CI 171*
				Guidance note for the Implementation of Standard Threshold Shift in The Medical Surveillance of Noise-Induced Hearing Loss 2016		*Follows CI 171*
Shipping	MSA	Maritime Occupational Safety Regulations, 1994	Nil	Safe Working Practices for Merchant Seamen		Exposure limit given as 87 dBA from The Merchant Shipping and Fishing Vessels (Control of Noise at Work) Regulations 2007, UK
				Safe Working Practices for Fishing Vessels		
				SA Ports Cargo Handling Code of Practice		Under OHSA and NIHLR
All	COIDA	*CI 171*		Schedule 3		Diseases caused by specified chemical and physical agents: by excessive noise by abnormal atmospheric or water pressure by ionising radiations
**Regulatory Law**						
***Civil Aviation Act*, No. 13 of 2009** This act under the Minister of Transport is aimed, *inter alia,* ‘to provide for the control and regulation of aviation within the Republic’ (Republic of South Africa, 2009). The Director of Civil Aviation, empowered by the CCA, issued the Civil Aviation Regulations (Republic of South Africa, 2011).						
**Regulations and standards**						
The Civil Aviation Regulations (Republic of South Africa, 2011).					These regulations incorporate the South African Civil Aviation Technical Standards (SA-CATS).	
The South African Civil Aviation Technical Standards (SA-CATS) (South African Civil Aviation Authority, 2011).					This document which contains ‘…the technical standards (that) contain the standards, rules and requirements which are applicable in respect of particular Parts of the (Civil Aviation) Regulations…’ (Republic of South Africa, 2011). The aviation industry has a system of medical fitness certification of personnel, which is specified in the SA-CATS 67. The requirements for the four classes of medical fitness certificates depending on occupational designation and a schedule of protocols for specific medical assessments are given. The Guide for Aviation Medical Examiners (South African Civil Aviation Authority, 2017) acknowledges the International Civil Aviation Organisation Manual of Civil Aviation Medicine (International Civil Aviation Organisation, 2012), which provides substantial information on medical examination protocols for aviation.	
***South African Maritime Safety Authority Act, 1998*** The authority by this act administers the MSA and its regulations and therefore is not under the jurisdiction of the OHSA (Republic of South Africa, 1993a).						
**Standard**						
The South African Maritime Safety Authority (SAMSA) has released the ‘Operations – Seafarer Certification, Guidance Note, Maritime Qualifications Code, The Maritime Medical Standards Code’ (South African Maritime Safety Authority, 2016).						
***National Railway Safety Regulation Act No 16 of 2002*** This act under the Department of Transport aims to set ‘safety standards and regulatory practices for the protection of persons, property and the environment’ (Republic of South Africa, 2002b).						
**Regulations**						
Via sections 28, 37 and 42 **The Railway Safety Standards Development Regulations, 2014 (Republic of South Africa, 2014a)**					These draft regulations incorporate the Southern African Railway Association (SARA) (2011) Human Factors Management into the legal framework of the Rail sector. ‘Annex D Medical action and exclusion criteria for employees who undertake safety-related-work’ provides medical fitness standards for assessment and evaluation (SARA, 2011).	
**Regulatory Law**						
***National Nuclear Regulator Act, 1999*** This act falls under the Minister of Energy and is purposed, *inter alia*, ‘to provide for safety standards and regulatory practices for protection of persons, property and the environment against nuclear damage’ (Republic of South Africa, 1999).						
**Regulations**						
Via section 47 **The Regulations on Safety Standards and Regulatory Practices 2006**					Require that a ‘comprehensive medical surveillance programme and health register must be established and maintained for all occupationally exposed workers’ (Republic of South Africa, 2006). In this regard, the following specifications for compliance have been developed to provide the details for routine medical testing: ‘The RD -011 Requirements for Medical Surveillance and Control of Persons Occupationally exposed to Radiation: Mining and Minerals Processing and the Requirements for Medical and Psychological Surveillance and Control at Koeberg Power Station’ (2004) and ‘LD -1077 The Requirements for Medical Surveillance and Control of Persons Occupationally exposed to Radiation: Mining and Minerals Processing’ (2002)	
**Regulations**						
Public Health Amendment Act, No. 42 of 1971 (Republic of South Africa, 1971). The ‘Regulations Concerning the Control of Electronic Products 1973’ requires ‘radiation workers’ to be certified medically fit (Republic of South Africa, 1973).						
***Hazardous Substances Act,*** **No. 15 of 1973** *The act aims to ‘provide for the control of substances which may cause injury or ill-health to or death of human beings’ (1973).*						
**‘Regulations Relating To Group IV 1993’ (Republic of South Africa, 1993a)**						

CAA, Civil Aviation Act; OHSA, Occupational Health and Safety Act; MHSA, Mine Health and Safety Act; MSA, Maritime Shipping Act; COIDA, Compensation for Occupational Injuries and Diseases Act; SA, South Africa; UK, the United Kingdom; NIHLR, noise-induced hearing loss regulations.

**TABLE A2:** Summary of results in regulatory law.

Department	Act	Regulations		Code of practice	Notes
Transport	CAA	Civil Aviation Regulations, 2011		SA CATS 67	Hearing loss at >35 dB at each frequency and vestibular dysfunction.
	SAMSA	Merchant Shipping (Eyesight And Medical Examination) Amendment Regulations, 2015		The Maritime Medical Standards Code	Vertigo is included in the Table of fitness criteria for common medical conditions
	NRSRA	Railway Safety Standards Development Regulations		SARA Human Factors Management Standard	Deterioration in hearing threshold of > 15 dB and vestibular dysfunction.
	NRTA	National Road Traffic Regulations, 2000		No standards for medical fitness SASOM Medical Requirements For Fitness To Drive – voluntary guideline	Has no standard for vestibular dysfunction
Energy	NNR	Regulation On Safety Standards And Regulatory Practices		RD - 011 The Requirements for Medical Surveillance and Control of Persons Occupationally Exposed to Radiation: Mining and Minerals Processing	No standards for medical fitness.
				LD -1077 Requirements for Medical and Psychological Surveillance and Control at Koeberg Power Station	No standards for medical fitness.
***Compensation Law***					
There are two compensation systems in South Africa which are governed by two different statutes: The *Compensation for Occupational Injuries and Diseases Act* (COIDA) and the *Occupational Diseases in Mines and Works Act* (ODMWA). These acts differ in their administration and the benefits provided.					
***Occupational Diseases in Mines and Works Act*, No. 78 of 1973** This act requires the monitoring, surveillance and evaluation of both former and active miners for specified occupational diseases only.					
***Compensation for Occupational Injuries and Diseases Act*, No. 130 of 1993** This act was promulgated in 1993 and administered by the erstwhile Department of Labour. The *COIDA* covers occupational injuries and diseases in all industries, including those from the mining sector that are not covered by ODMWA, as listed in Schedule 3 (Republic of South Africa, 1993c).					
**Circular**					
**The Compensation Commissioner’s ‘Circular Instruction No.171 and Supplement entitled Determination of Permanent Disablement Resulting from Noise-Induced Hearing Loss and Trauma’ (Department of Labour, 2001).**			Outline procedure to be followed for the claiming of compensation for noise-induced hearing loss.		

CAA, Civil Aviation Act; SAMSA, South African Maritime Safety Authority Act; NRSRA, National Rail Safety Regulator Act; NRTA, National Road Traffic Act; NNR, National Nuclear Regulator Act.
